# A Rare Case of Diffuse Alveolar Hemorrhage Caused by Fentanyl Inhalation

**DOI:** 10.7759/cureus.47598

**Published:** 2023-10-24

**Authors:** Aneeta Nangrani, Justin Chung, Edison Jyang, Kunal Nangrani, Marie Schmidt

**Affiliations:** 1 Pulmonary and Critical Care Medicine, One Brooklyn Health, Brooklyn, USA; 2 Internal Medicine, State University of New York Downstate Medical Center, Brooklyn, USA; 3 Internal Medicine, Medical University of Lublin, Lublin, POL; 4 Pulmonary and Critical Care Medicine, Mount Sinai Hospital, Queens, USA; 5 Pulmonary and Critical Care Medicine, Interfaith Medical Center, Brooklyn, USA

**Keywords:** opioid, diffuse alveolar hemorrhage, fentanyl, pulmonary hemorrhage, dah

## Abstract

Diffuse alveolar hemorrhage (DAH) is a rare but life-threatening pulmonary disorder characterized by blood accumulation in alveolar spaces, often associated with autoimmune diseases and infections. Drug-induced causes of DAH, including inhalation of substances like fentanyl, are emerging concerns. A 40-year-old male with bipolar disorder and polysubstance abuse presented with altered mental status and hemoptysis after inhaling an unknown substance. Physical examination revealed respiratory distress, pinpoint pupils, and severe hypoxemia. Naloxone administration improved his condition. The workup showed negative infection markers, positive fentanyl-specific urine test, and diffuse bilateral opacities on imaging. Bronchoalveolar lavage confirmed DAH with >20% hemosiderin-laden macrophages. Steroid treatment resulted in marked improvement. Drug-induced DAH, such as fentanyl inhalation, should be considered in patients with altered mental status and pulmonary symptoms following substance use. Comprehensive evaluation and targeted treatment are crucial for optimal outcomes.

## Introduction

Diffuse alveolar hemorrhage (DAH) is a life-threatening medical emergency characterized by the accumulation of blood within the alveolar spaces of the lungs, leading to impairment of gas exchange and, in worst-case scenarios, posing a life-threatening respiratory compromise. While DAH is most frequently associated with autoimmune diseases - such as granulomatosis with polyangiitis or microscopic polyangiitis - infections, and coagulation disorders as its primary triggers, it is of paramount importance to recognize the role played by ascendant drug-induced factors in its etiology [1].

Fentanyl, a synthetic opioid renowned for its high potency and analgesic properties, has gained significant clinical importance in pain management, procedural sedation, and general anesthesia. Its efficacy and favorable pharmacokinetic profile have led to its extensive use across various medical settings. While it is traditionally administered via intravenous or transdermal routes, in recent years, fentanyl inhalation has emerged as an alternative method of delivery, particularly in the context of recreational drug use. Fentanyl powder can be inhaled knowingly or unknowingly via other recreational drugs, which have been contaminated with the opioid. Considering this, the potential for adverse effects associated with fentanyl inhalation is an area of growing concern. Amongst those risks, DAH is present, with rare cases of fentanyl-induced DAH being observed [2,3]. This case presents a patient with DAH after reporting the inhalation of an unknown substance, subsequent to which testing confirmed the presence of cannabis and fentanyl.

## Case presentation

A 40-year-old male with a medical history notable for bipolar disorder, polysubstance abuse, and a previous diagnosis of Guillain-Barre syndrome presented to the emergency department with an altered mental status and hemoptysis. The patient reported inhaling an unknown substance, which prompted his clinical presentation. Upon physical examination, the patient exhibited respiratory distress, bilateral pulmonary rales, and pinpoint pupils bilaterally. Vital signs revealed a blood pressure of 130/75 mmHg, heart rate of 111 beats per minute, temperature of 36.6°C, a respiratory rate of 20 breaths per minute, and a pulse oximetry (SpO2) reading of 80% on room air.

Prompt administration of naloxone 0.4 mg in the ED resulted in a positive response, improving the patient's mental status and respiratory function. Urine toxicology was positive only for cannabinoids. Given the patient's positive reaction to naloxone and the elevated clinical suspicion arising from the escalating prevalence of drug products adulterated with fentanyl, a specialized fentanyl-specific urine test was obtained and confirmed a positive result. Nevertheless, because of the severity of the initial presentation, the patient was subsequently admitted to the ICU for the management of acute toxic metabolic encephalopathy and acute hypoxic hypercarbic respiratory failure.

**Figure 1:**
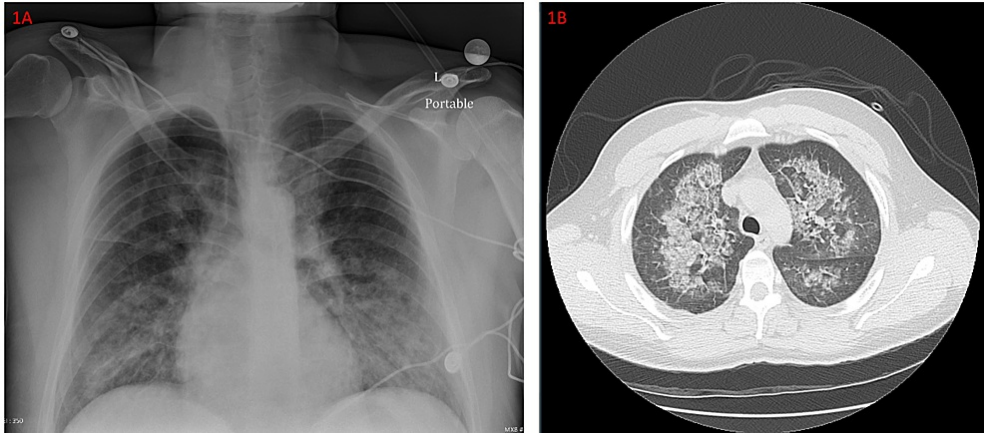
A chest radiograph on admission shows diffuse bilateral interstitial opacities (1A). The patient’s CT chest on admission shows diffuse bilateral ground-glass opacities (1B).

An arterial blood gas analysis upon arrival showed acute respiratory acidosis with a pH of 7.16, PCO2 of 73.7 mmHg, and PaO2 of <45 mmHg. Complete blood count, comprehensive metabolic panel, and urinalysis were unremarkable (Table 1). The patient's workup for COVID-19, influenza, legionella, mycoplasma, and HIV all returned negative results. Blood cultures were also negative. Autoimmune testing was negative. Normal sinus rhythm was seen on an electrocardiogram, with negative troponins and a normal brain natriuretic peptide of 66 pg/mL. Both chest X-ray and computed tomography (CT) scan showed diffuse bilateral opacities (Figure 1). At that time, the differential diagnoses included acute respiratory distress syndrome, pulmonary edema, multilobar pneumonia, aspiration pneumonitis, acute eosinophilic pneumonia, and DAH.

**Table 1:** An overview of laboratory work-up and results. Reference ranges and/or units are provided in parentheses. WBC: White blood cells; RBC: Red blood cells; HGB: Hemoglobin; HCT: Hematocrit; MCV: Mean corpuscular volume; MCHC: Mean corpuscular hemoglobin concentration; RDW: Red cell distribution width; CO2: Carbon dioxide; BUN: Blood urea nitrogen; ALP: Alkaline phosphatase; AST: Aspartate transaminase; ALT: Alanine transaminase; PT: Prothrombin time; PTT: Partial thromboplastin time; INR: International normalized ratio; BAL: Bronchoalveolar lavage

Complete Blood Count		Comprehensive Metabolic Panel	
WBC (4.5-11.0 x 10^9 cells/L)	10.8	Sodium (135-145 mEq/L)	138
RBC (4.3-5.9 million cells/mm^3)	5.02	Potassium (3.5-5.1 mEq/L)	3.8
HGB (13.5-17.5 g/dL)	12.7	Chloride (96-106 mmol/L)	105
HCT (41-53%)	38.9	CO2 (20-29 mmol/L)	22
MCV (80-100 µm^3)	77.5	BUN (7-20 mg/dL)	17
MCH (25.4-34.6 pg/cell)	25.3	Creatinine (0.7-1.3 mg/dL)	1.2
MCHC (31-36% Hb/cell)	32.6	Glucose (70-100 mg/dL)	312
RDW (12-15%)	13.9	Albumin (3.9-5.0 g/dL)	4.1
Platelets (150-400/mm^3)	318	ALP (44-147 IU/L)	101
BAL Fluid Analysis		AST (10-34 IU/L)	44
Color (Clear)	Red	ALT (8-37 IU/L)	33
WBC (cells/µL)	213	Total Bilirubin (0.1-1.2 mg/dL)	0.6
RBC (<0 cells/µL)	6,875	Coagulation Profile	
Neutrophil (%)	71	PT (11-13.5 s)	10.3
Lymphocyte (%)	17	PTT (25-36 s)	25.1
Monocyte (%)	8	INR (0.8-1.1)	0.87
Macrophages (%)	4

**Figure 2:**
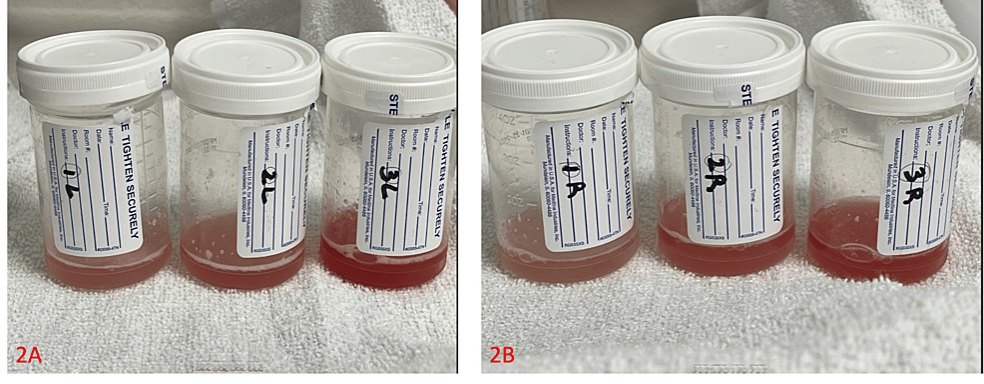
Fluid collected from the left lung (2A) and right lung (2B) during a BAL becomes bloodier over three sequential aliquots.

**Figure 3:**
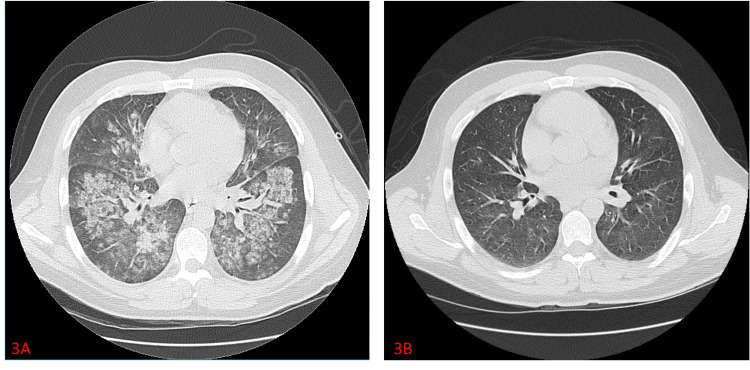
CT scan of the patient’s chest before (3A) and after corticosteroid treatment (3B), showing marked improvement from previous diffuse ground-glass opacities.

Subsequently, a flexible bronchoscopy with bronchoalveolar lavage (BAL) was performed, which yielded red, bloody fluid in three consecutive sets of aliquots (Figure 2). A culture was positive for likely contaminants of rare candida species. Malignant cells were not detected in the cytology, but it did reveal 57% of cells to be hemosiderin-laden macrophages, which is indicative of DAH. The patient was initiated on pulse-dose methylprednisolone 1 g daily for five days, followed by an oral tapering. A repeat CT scan of the chest following five days of steroid treatment revealed marked improvement (Figure 3).

## Discussion

As the recreational use of inhaled fentanyl has surged in popularity - both knowingly and unknowingly - it is imperative to understand the myriad adverse effects this substance can inflict upon the human body. This case serves as an illustration of the connection between the inhalation of fentanyl and the occurrence of DAH. As evidenced in this case, it is worth noting that, while hemoptysis traditionally serves as a hallmark manifestation of DAH, it can be absent in approximately 33% of cases [4]. Therefore, the absence of hemoptysis should not preemptively dismiss the possibility of DAH in a differential diagnosis, especially when there exists a high clinical suspicion. The triad of impaired gas exchange, respiratory distress, and hemoptysis observed in this case, coupled with the bronchoalveolar lavage findings, aligns with the typical clinical manifestations associated with DAH.

Various mechanisms have been proposed to explain opioid-induced DAH. One theory implicates a surge in catecholamines triggered by opioid withdrawal following naloxone administration. This surge in hormonal activity not only heightens cardiac afterload and strain but also promotes alveolar fluid accumulation, potentially resulting in direct injury to pulmonary endothelial cells because of pulmonary circulation constriction [2,5,6]. Another theory suggests opioid-induced DAH may be linked to negative pressure barotrauma, stemming from attempts to inhale against a closed glottis during opioid exposure [7,8]. Based on this theory, a deep inhalation following naloxone administration can exacerbate the pressure gradient, culminating in alveolar fluid accumulation, acute lung injury, and possibly DAH.

Timely recognition and accurate diagnosis of DAH are paramount for instituting appropriate management strategies. Nevertheless, the intricacies of this case underscore the diagnostic challenges of opioid-induced DAH, requiring a comprehensive evaluation to eliminate alternative causes of the patient's presentation. Diagnostic tools such as bronchoscopy with bronchoalveolar lavage and various imaging studies may be deployed to confirm the diagnosis and exclude other potential etiologies. Laboratory findings for DAH can be nonspecific, and, thus, diagnostic testing typically focuses on assessing potentially related symptoms, such as bleeding disorders, or concurrent kidney disease, while also considering historical and physical examination clues regarding underlying causes. Additionally, in cases of suspected DAH, it is imperative to meticulously explore other possible infectious etiologies, including bacterial pneumonia and COVID-19 [9].

Given the broad nature of differential diagnosis for DAH, initial treatment should be empiric and cover a wide spectrum of possibilities. Once a diagnosis of DAH is confirmed, targeted therapy can be initiated. Antibiotics may be considered if infection is suspected or confirmed, although their routine use is not indicated in the absence of clear infection evidence. Glucocorticoids, such as methylprednisolone or prednisone, play a pivotal role in DAH management, particularly when associated with autoimmune or systemic vasculitic conditions. These medications aim to mitigate the inflammatory process and stabilize pulmonary vasculature [4,10].

Extracorporeal membrane oxygenation (ECMO) represents a potential treatment modality that has been utilized in select cases of refractory hypoxemic respiratory failure because of DAH [11]. However, the role of ECMO in DAH management remains limited and contentious because of the necessity for anticoagulation, which may exacerbate alveolar hemorrhage [12]. Some reports suggest the avoidance of anticoagulation during ECMO; nevertheless, these patients remain at a high risk of thromboembolism [13-15].

## Conclusions

This case report emphasizes the link between inhaled fentanyl and DAH and calls for heightened clinical vigilance because of the rising recreational fentanyl use. It underscores the diverse clinical presentation of DAH and the need for comprehensive evaluation. Further research is required to understand the mechanisms behind drug-induced DAH and improve patient care.
